# On the Development of All-Cellulose Capsules by Vesicle-Templated Layer-by-Layer Assembly

**DOI:** 10.3390/polym13040589

**Published:** 2021-02-16

**Authors:** Alireza Eivazi, Bruno Medronho, Björn Lindman, Magnus Norgren

**Affiliations:** 1 FSCN, Surface and Colloid Engineering, Mid Sweden University, SE-851 70 Sundsvall, Sweden; bfmedronho@ualg.pt (B.M.); magnus.norgren@miun.se (M.N.); 2 MED—Mediterranean Institute for Agriculture, Environment and Development, Universidade do Algarve, Faculdade de Ciências e Tecnologia, Campus de Gambelas, Ed. 8, 8005-139 Faro, Portugal; 3 Physical Chemistry, University of Lund, P.O. Box 124, S-221 00 Lund, Sweden; bjorn.lindman@fkem1.lu.se; 4 Department de Quìmica, University of Coimbra, Rua Larga, 3004-535 Coimbra, Portugal

**Keywords:** didodecyldimethylammonium bromide, vesicle template, Layer-by-Layer (LbL), carboxymethylcellulose, quaternized hydroxyethylcellulose ethoxylate, cellulose capsules

## Abstract

Polymeric multilayer capsules formed by the Layer-by-Layer (LbL) technique are interesting candidates for the purposes of storage, encapsulation, and release of drugs and biomolecules for pharmaceutical and biomedical applications. In the current study, cellulose-based core-shell particles were developed via the LbL technique alternating two cellulose derivatives, anionic carboxymethylcellulose (CMC), and cationic quaternized hydroxyethylcellulose ethoxylate (QHECE), onto a cationic vesicular template made of didodecyldimethylammonium bromide (DDAB). The obtained capsules were characterized by dynamic light scattering (DLS), ζ potential measurements, and high-resolution scanning electron microscopy (HR-SEM). DLS measurements reveal that the size of the particles can be tuned from a hundred nanometers with a low polydispersity index (deposition of 2 layers) up to micrometer scale (deposition of 6 layers). Upon the deposition of each cellulose derivative, the particle charge is reversed, and pH is observed to considerably affect the process thus demonstrating the electrostatic driving force for LbL deposition. The HR-SEM characterization suggests that the shape of the core-shell particles formed is reminiscent of the spherical vesicle template. The development of biobased nano- and micro-containers by the alternating deposition of oppositely charged cellulose derivatives onto a vesicle template offers several advantages, such as simplicity, reproducibility, biocompatibility, low-cost, mild reaction conditions, and high controllability over particle size and composition of the shell.

## 1. Introduction

The development of nanostructured materials (i.e., inorganic, organic, polymeric, and biological) with different composition, morphology, and sizes is of great scientific and technological interest due to their huge potential in many areas ranging from the encapsulation and controlled release of drugs, protection of biologically active species, and removal of pollutants, to the development of advanced materials suitable for cosmetics, inks, or catalysis [1,2,3,4,5]. These systems can be synthesized with well-defined structural features conferring control over different properties, such as mechanical, optical, electrical, and biological, among others [6]. In this respect, the Layer-by-Layer (LbL) assembly is, most likely, the most versatile approach to assemble multifunctional nanoscale materials with precise control regarding composition and structure [7,8,9,10,11,12,13]. Thus, through alternating adsorption of cationic and anionic macromolecules, well-defined surface layers with variable thickness (from a few to hundreds of Å) can be created. Both films on solid surfaces, free-standing films, and hollow capsules can be prepared. Gero Decher was the first to present the LbL principle [14,15,16,17,18,19]. Earlier, Iler had presented an approach of alternating adsorption of cationic and anionic colloidal particles (e.g., silica, alumina, etc.) on a glass surface [20]. Iler’s approach is conceptually interesting, but of limited use since the layers are not very stable and do not have molecular dimensions.

Among principles for preparation of well-defined surface layers, the LbL technique continues to have a very strong position. The original studies regarding LbL concerned macroscopic surfaces and this has continued to be the main line in research and applications. Excellent reviews on the LbL as well as their different applications can be found elsewhere [9,21,22,23,24,25,26,27,28,29,30,31,32,33,34].

Polyelectrolyte-based systems are the most commonly used, but the LbL technique is also applicable to almost any other type of charged systems, including inorganic molecular clusters, nanoparticles [35], nanotubes [36,37], nanoplates [38], micelles [39,40], block copolymers [41], polysaccharides [42], polypeptides [43], DNA [44], and viruses [45]. These can be successfully incorporated as components to prepare LbL films [46]. Moreover, beyond the typical use of polymers in the LbL assembly, even hybrid LbL materials can be developed intercalating in the layered films of other structures, such as carbon nanotubes [47], or even biomolecules [48].

The LbL concept was extended in a very interesting way by Möhwald and coworkers, who started research on deposition onto colloidal particles (nanoparticles), for example inorganic particles (like silica and CaCO3) and polymer particles, but also emulsion droplets and biological particles like virus and cells [5,27,49,50,51,52]. By dissolution of the particle, not only can core-shell particles, but also hollow capsules, be prepared (with sizes from a few hundreds of nanometer). For the preparation of hollow capsules, a critical step is the removal of the core particle and, in most studies, this required rather harsh conditions (like concentrated hydrofluoric acid). As a way of circumventing this problem, Cuomo et al. have introduced surfactant vesicles as a template; by a simple variation in the solvency conditions, for example by adding a non-ionic surfactant, the vesicle-forming surfactant can be easily removed [53]. Thus, Cuomo et al. have developed a mild method to obtain hollow nano-sized structures via the alternating deposition of oppositely charged alginate and chitosan on a dimethyldioctadecylammonium bromide (DDAB)-based vesicular template [53,54]. With such an approach, the use of strong acids or other harsh conditions can be avoided, ensuring the capsule integrity. Successful biopolymer systems include mixtures, such as chitosan and fucoidan [55] or chitosan and lignosulfonate [56]. Biocompatible and thermo-responsive nanocapsule templating, using DDAB-based vesicles, has also been reported [57]

The use of synthetic polyelectrolyte complexes has been described in detail and reviewed [58]. The use of natural polysaccharides represents an attractive alternative for practical and biomedical applications due to their sustainability, biocompatibility, and biodegradability properties [1,59,60]. In this respect, biopolymers such as alginate and chitosan have been widely investigated for many applications including the development of LbL nanocapsules [53,59,61,62]. Cellulose, as the most available biopolymer on the planet, deserves to be highlighted. The formation of LbL self-assemblies using combinations of nanocelluloses (both anionic and cationic) and polyelectrolytes looks very appealing and has been recently reviewed [63].

Oppositely charged cellulose derivatives, such as anionic carboxymethylcellulose (CMC), and cationic quaternized hydroxyethylcellulose ethoxylate (QHECE), have been successfully deposited on silicon or inorganic quartz substrates forming thin films [64]. To our knowledge, the use of cellulose derivatives in vesicular templates has never been reported before. In this work, the feasibility of LbL particle formation, using two oppositely charged cellulose derivatives, CMC and QHECE, is demonstrated on a DDAB vesicle template. The obtained nanospheres are characterized by dynamic light scattering (DLS), the ζ potential, and high-resolution scanning electron microscopy (HR-SEM).

## 2. Materials and Methods

### 2.1. Materials

The cellulose derivatives carboxymethylcellulose, CMC (degree of substitution of 0.65–0.90; viscosity of a 1% aqueous solution is 1500–3000 cps), and quaternized hydroxyethylcellulose ethoxylate, QHECE, (viscosity of a 1% aqueous solution is 300–500 cps), the cationic surfactant didodecyldimethylammonium bromide, DDAB (purity of 98%), NaOH (≥98%), and HCl (30%) were purchased from Sigma-Aldrich. Deionized water was used in the preparation of all solutions.

### 2.2. Stock Solutions of Vesicles and Cellulose Derivatives

DDAB spontaneously forms vesicles in very dilute solutions as reported elsewhere [65,66]. To produce the vesicle template, a stock solution of DDAB was prepared by dissolving 0.23 g of DDAB in 50 mL of deionized water (50 mL vial). The solution was then carefully stirred at room temperature with a magnetic stirrer (200 rpm) until the surfactant was fully dissolved (ca. 4 h), and freshly used in the experiments. The stock solutions of CMC and QHECE were prepared by dissolving 50 mg of each cellulose derivative in 50 mL deionized water. The solutions were vigorously stirred (300 rpm) at room temperature until the dissolution was achieved (ca. 24 h). Regarding the pH effect, NaOH and HCl were used to adjust the pH with a Mettler Toledo pH meter (Model MP 225) calibrated for the studied pH range.

### 2.3. Layer-by-Layer Deposition

**Figure 1:**
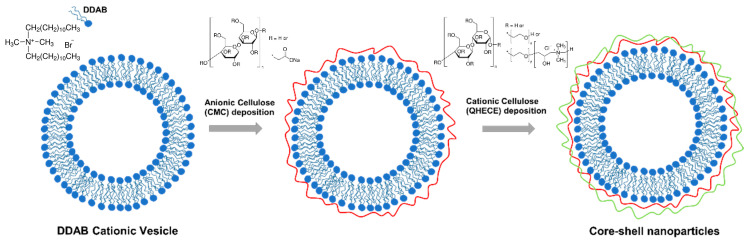
Schematic representation of the alternating LbL deposition of anionic carboxymethylcellulose (CMC) and cationic quaternized hydroxyethylcellulose ethoxylate (QHECE) unto a cationic dimethyldioctadecylammonium bromide (DDAB) vesicle.

The DDAB cationic vesicles were coated with alternating layers of negatively charged CMC and positively charged QHECE, following the procedure described by Cuomo et al. [53] and schematically represented in Figure 1. Briefly, the amount of each cellulose derivative for the formation of the individual shells was established through titration, adding each polyelectrolyte solution until the isoelectric point was exceeded; this corresponds to a higher instability of the aggregates due to the overall low charge density. Samples were visually checked for turbidity changes, and the achievement of the isoelectric point was followed by measuring the ζ potential of the surface that results close to the neutrality. A minor increment of the added volume of the polyelectrolyte results in the reverse surface charge. Beyond the isoelectric point, the surface was completely covered with a certain cellulose derivative and the electrostatic repulsive forces between the aggregates ensured the stability of the complexes. After the deposition of each layer, the suspensions were centrifuged at 25 °C in two steps; 1500 rpm for 15 min, and 2000 rpm for 15 min. With this sequential centrifugation approach, the recovery of the supernatant containing more monodisperse and electrostatically stable coated vesicles (without unstable aggregates, which may form during deposition of each layer of studied cellulose derivatives) is more efficient. Thereafter, the collected suspension was fully characterized and used for further layer deposition.

### 2.4. Characterization Methods

The average hydrodynamic diameter and the ζ-potential values of the aggregates were determined by means of dynamic light scattering measurements and electrophoretic mobilities by laser Doppler velocimetry, respectively, using a Malvern UK Zetasizer-Nano ZS90 commercial instrument operating with a 4 mW He-Ne laser (633 nm wavelength). The average aggregate size was estimated with a fixed detector angle of 90° by a cumulant analysis of the autocorrelation function using the software provided by the manufacturer. ζ-potential measurements were performed by checking the electrophoretic mobilities of the aggregates as determined by laser Doppler velocimetry. The detection angle was 17°. Samples were injected into dedicated disposable capillary cells. All measurements were performed at 25 °C, and each sample was measured in triplicate. The mean values are reported with a relative standard deviation lower than 10%.

Particles were imaged with a high-resolution scanning electron microscope (HR-SEM), TESCAN MAIA3 model 2016. Briefly, the samples were deposited on a silica surface and, after air-dried, they were coated with ca. 2 nm thick layer of iridium (Q150 T ES; Quorum Technologies, Lewes, UK) to obtain an electrically conductive surface. Secondary electron images (SEI) were generated using 5 kV accelerating voltage and an in-lens detector.

## 3. Results

**Figure 2:**
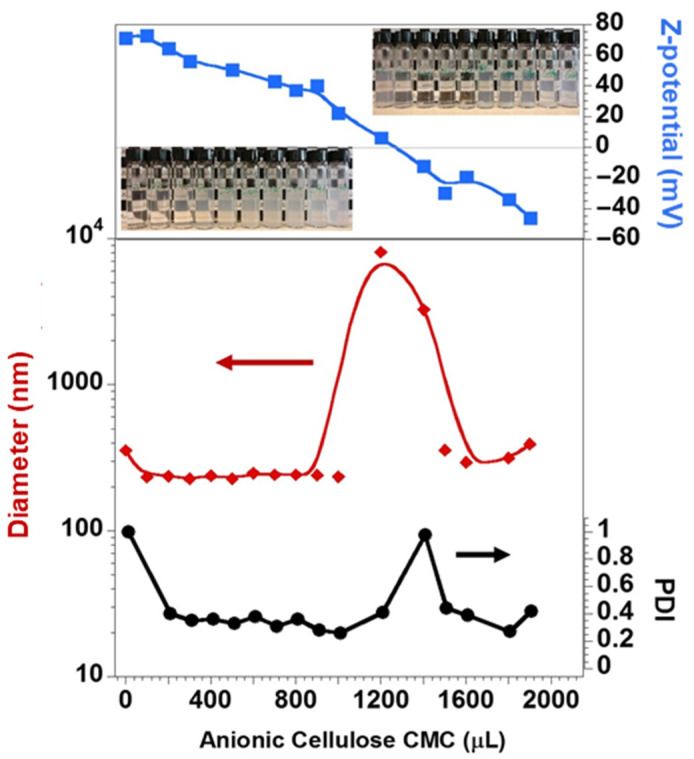
Changes of the average size, PDI, and ζ-potential of the DDAB vesicles upon the progressive addition of CMC. The average values are reported with a relative standard deviation lower than 3%. The inserted photos show the evolution of turbidity of the samples with CMC addition. Lines are just guides for the eyes.

Cationic DDAB vesicles were used as the soft colloidal template for the LbL deposition of negatively charged CMC and positively charged QHECE. The average diameter and the surface charge of the bare vesicles were 355 nm and +70.9 mV, respectively. As described in the experimental section, the cationic vesicles were first covered with increasing amounts of the anionic CMC (keeping constant the vesicle concentration) until the isoelectric point was reached. As can be seen in Figure 2, such progressive titration gradually decreases the surface charge density of the particles. Near the isoelectric point, samples are visually more turbid (photos inserted in Figure 2) and the average size and polydispersity index increased considerably. Following this instability region, the charge density of the particles is reversed, and the deposition was considered completed when the average size and PDI stabilized. After the first layer of CMC, the particles presented an average size of 306 nm (i.e., 303, 305, and 310 nm) and a surface charge of −45 mV (i.e., −43, −44, and −48 mV).

**Figure 3:**
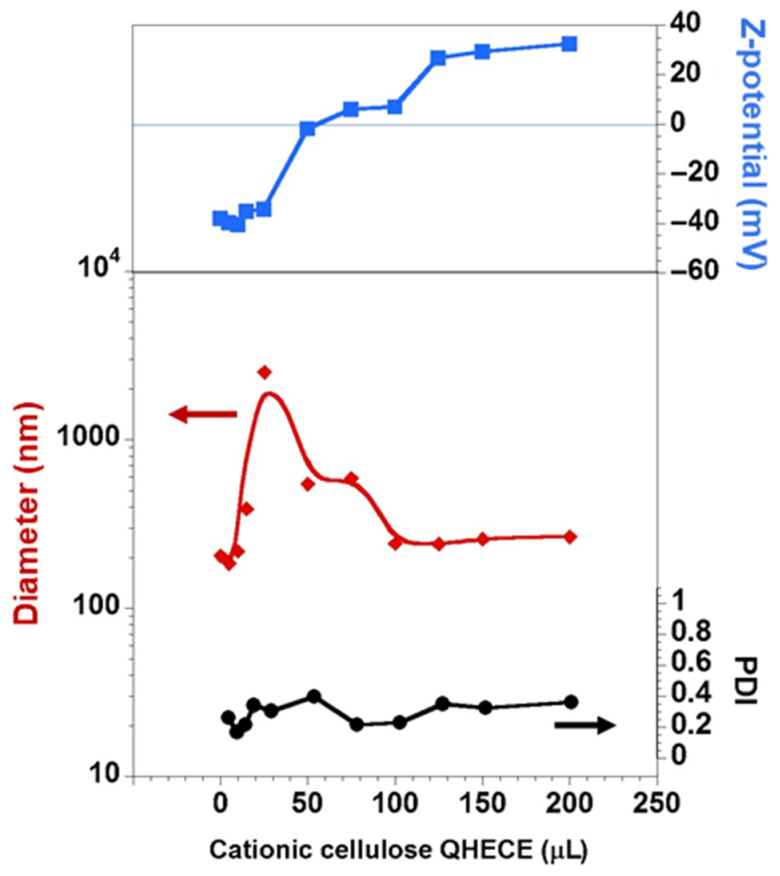
Changes of the average size, PDI, and ζ-potential of the DDAB/CMC vesicles upon the progressive addition of QHECE. The average values are found to have a relative standard deviation lower than 3%. Lines are just guides for the eyes.

The same procedure was followed for the second layer deposition (i.e., the cationic QHECE). As can be seen in Figure 3, the addition of increasing amounts of QHECE progressively increases the ζ-potential. Similarly to the CMC first layer deposition, upon reaching the isoelectric point, the samples become more unstable, and particles of larger size are formed. Finally, further additions of the positively charged polyelectrolyte reverse again the surface charge and particles with an average size of 266 nm (i.e., 268, 257, and 273 nm) and a net surface charge of +32.5 mV (i.e., +32, +32, and +33.5 mV), are formed.

**Figure 4:**
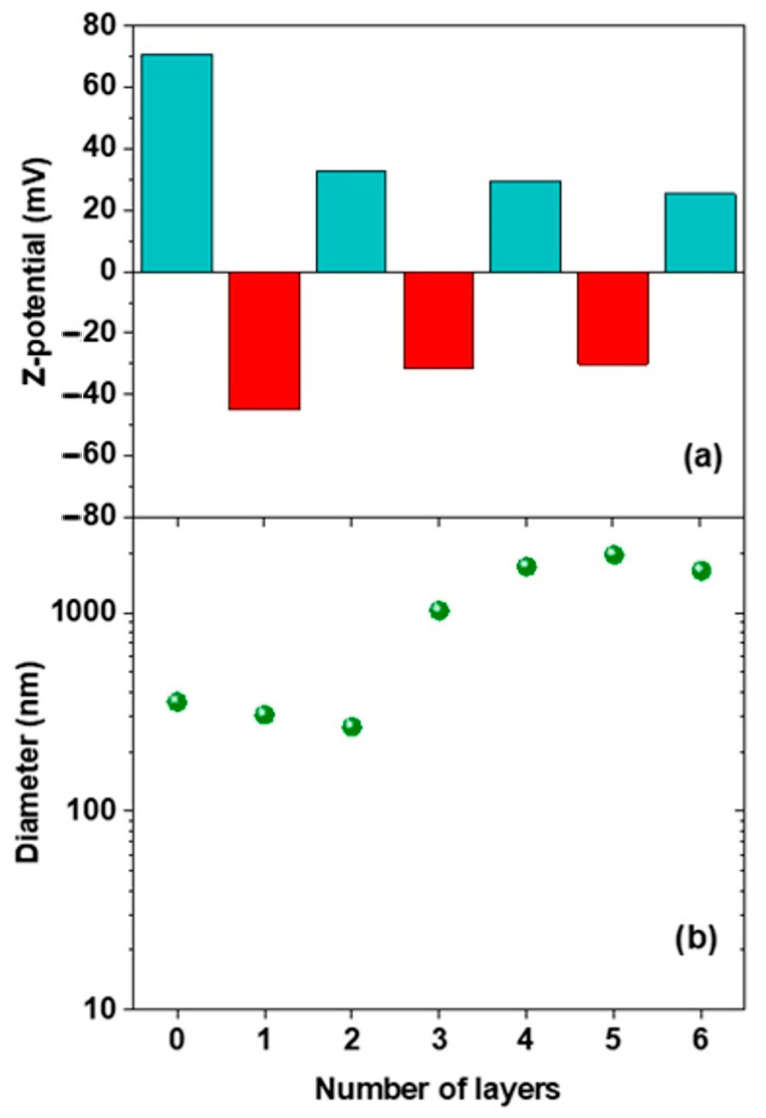
The dependence of the ζ-potential (**a**) and particle size (**b**) on the cellulose derivative type and number of layers deposited. The first values in both panels refer to the bare DDAB vesicle. The average standard deviation is lower than 5% for the first 3 layers and below 10% for the subsequent four to six layers.

The procedure described above was sequentially applied for the following deposition of CMC and QHECE layers, and in Figure 4 the surface charge and size of the formed particles are shown as a function of the number of adsorbed layers.

Depending on the deposited polyelectrolyte the particles formed display either a positive or a negative surface net charge with values ranging between −45 mV and +33 mV. Furthermore, the mean size of the aggregates progressively increases with the addition of the polyelectrolyte layers, from 306 nm (1st layer) up to 1600 nm (6th layer).

**Figure 5:**
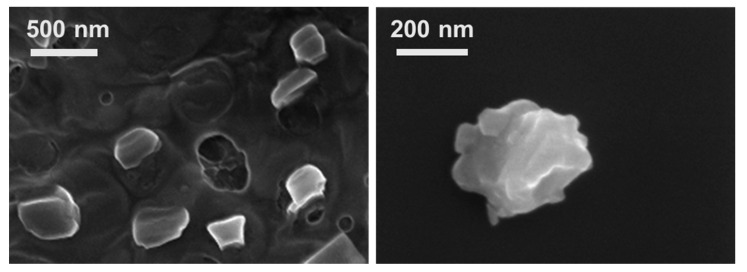
HR-SEM images of cellulose core-shell particles formed after the first deposition of the anionic cellulose derivative CMC onto the DDAB vesicles at 69 and 200 kx, respectively.

The aggregates formed were further analyzed by SEM and a typical example is displayed in Figure 5 for two different magnifications. Overall, the shape of the formed core-shell particles is reminiscent of the spherical DDAB vesicle template [53]. The observed deformations are a consequence of the pre-treatment drying procedure required for SEM analysis; since the water inside the vesicle templates is eliminated during drying, the structure is expected to partially collapse, but mainly retaining its spherical features.

**Figure 6:**
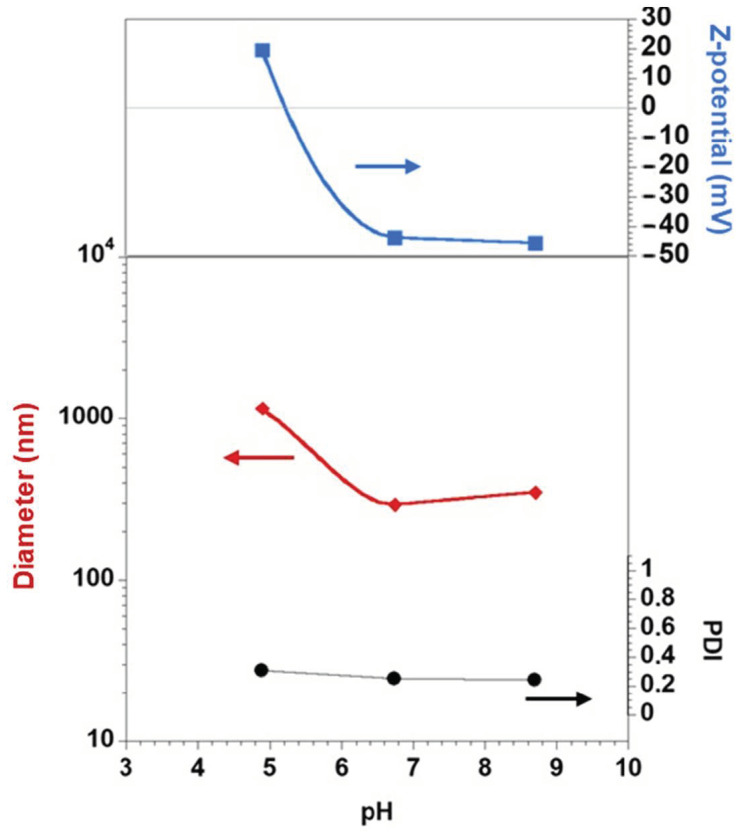
Evolution of the DDAB vesicles surface charge, PDI, and mean size with the deposition of CMC (1 g/L) at different pHs (i.e., 4.9, 6.75, and 8.7) at 25 °C. The average values are found to have a relative standard deviation lower than 3%. Lines are just guides for the eyes.

The adsorption of a charged cellulose derivative onto an oppositely charged vesicular template is expected to be caused by electrostatic interactions and mainly driven by the entropic gain due to release of counterions. This is exemplified in Figure 6 where the deposition of CMC onto the DDAB vesicle can be seen to strongly depend on pH. At the lowest pH value tested (i.e., 4.9), CMC is expected to be only partially ionized (pKa is 4.3) and therefore the interaction with DDAB vesicles is weaker than at higher pHs (i.e., 6.75 and 8.7) where the carboxylic groups are expected to be fully deprotonated. Since the same CMC concentration was used in all cases, the system with the lowest pH (and low degree of ionization of CMC) only slightly adsorbs and moderately decreases the net charge of the DDAB vesicles (from the neat +70.9 mV to +20 mV). This charge reduction of the vesicles is sufficient to make them less stable and prone to aggregate as demonstrated by the large size of the particles obtained. On the other hand, when the pH of the CMC solutions is far above the pKa, the cellulose derivative is expected to be fully deprotonated being capable to efficiently absorb onto the DDAB vesicles and reverse the charge. The electrostatic repulsion among the now negatively charged DDAB-CMC particles makes the system quite stable with a low PDI and mean size. The same electrostatically driven adsorption mechanism is expected for QHECE deposition.

## 4. Conclusions

In this work, novel biobased particles were successfully formed via a LbL approach; two cellulose derivatives, anionic CMC, and cationic QHECE, were alternatively deposited onto a soft cationic DDAB vesicle template. With the developed approach it was possible to tune the size and net charge of the all-cellulose core-shell particles. The driving force for adsorption is essentially electrostatic and, thus strongly pH dependent. The electron microscopy shows core-shell particles with a shape reminiscent of the spherical vesicle template. This simple and reliable approach can be expanded and consider chemical functionalization or the inclusion of other compounds for specific and target properties. Therefore, the development of nano and micro cellulose-based multilayer containers, using a simple and inexpensive LbL approach, paves the way to novel advanced cellulose-based materials and/or hybrid systems of superior features. Besides the opportunity to tune the charge of the particles and their dimensions, the importance of this work lies in the fact that the developed vesicle-template strategy can be readily extended to other liposome-based systems.

## Data Availability

Data are contained within the article. Additional data may be provided upon request.

## References

[1] Zhao Q., Han B., Wang Z., Gao C., Peng C., Shen J. (2007). Hollow chitosan-alginate multilayer microcapsules as drug delivery vehicle: Doxorubicin loading and in vitro and in vivo studies. Nanomed. Nanotechnol. Biol. Med..

[2] Del Mercato L.L., Rivera-Gil P., Abbasi A.Z., Ochs M., Ganas C., Zins I., Sönnichsen C., Parak W.J. (2010). LbL Multilayer Cap-sules: Recent Progress and Future Outlook for Their Use in Life Sciences. Nanoscale.

[3] Sukhorukov G.B., Möhwald H. (2007). Multifunctional cargo systems for biotechnology. Trends Biotechnol..

[4] Zhang Y., Huang Z., Tang F., Ren J. (2006). Ferrite hollow spheres with tunable magnetic properties. Thin Solid Films.

[5] Skirtach A.G., Dejugnat C., Braun D., Susha A.S., Rogach A.L., Parak W.J., Möhwald H., Sukhorukov G.B. (2005). The Role of Metal Nanoparticles in Remote Release of Encapsulated Materials. Nano Lett..

[6] Amaratunga G.A.J., Chhowalla M., Kiely C.J., Alexandrou I., Aharonov R., Devenish R.M. (1996). Hard elastic carbon thin films from linking of carbon nanoparticles. Nat. Cell Biol..

[7] Tang Z., Wang Y., Podsiadlo P., Kotov N.A. (2006). Biomedical Applications of Layer-by-Layer Assembly: From Biomimetics to Tissue Engineering. Adv. Mater..

[8] Ariga K., Hill J.P., Ji Q. (2007). Layer-by-layer assembly as a versatile bottom-up nanofabrication technique for exploratory research and realistic application. Phys. Chem. Chem. Phys..

[9] Wang Y., Angelatos A.S., Caruso F. (2008). Template Synthesis of Nanostructured Materials via Layer-by-Layer Assembly. Chem. Mater..

[10] Richardson J.J., Cui J., Björnmalm M., Braunger J.A., Ejima H., Caruso F. (2016). Innovation in Layer-by-Layer Assembly. Chem. Rev..

[11] Caruso F., Caruso R.A., Möhwald H. (1998). Nanoengineering of Inorganic and Hybrid Hollow Spheres by Colloidal Templating. Science.

[12] Caruso F., Lichtenfeld H., Donath E., Möhwald H. (1999). Investigation of Electrostatic Interactions in Polyelectrolyte Multilayer Films: Binding of Anionic Fluorescent Probes to Layers Assembled onto Colloids. Macromolecules.

[13] Caruso F., Donath E., Möhwald H. (1998). Influence of Polyelectrolyte Multilayer Coatings on Förster Resonance Energy Transfer between 6-Carboxyfluorescein and Rhodamine B-Labeled Particles in Aqueous Solution. J. Phys. Chem. B.

[14] Decher G. (1997). Fuzzy Nanoassemblies: Toward Layered Polymeric Multicomposites. Science.

[15] Decher G., Hong J.D. (1991). Buildup of Ultrathin Multilayer Films by a Self-Assembly Process: II. Consecutive Adsorption of Anionic and Cationic Bipolar Amphiphiles and Polyelectrolytes on Charged Surfaces. Ber. Bunsenges. Phys. Chem..

[16] Decher G.D., Hong J.-D. (1991). Buildup of ultrathin multilayer films by a self-assembly process, 1 consecutive adsorption of anionic and cationic bipolar amphiphiles on charged surfaces. Makromolekulare Chemie. Macromolecular Symposia.

[17] Decher G., Schmitt J., Helm C., Lösche M., Möhwald H. (1992). Fine-Tuning of the Film Thickness of Ultrathin Multilayer Films Composed of Consecutively Alternating Layers of Anionic and Cationic Polyelectrolytes. Trends in Colloid and Interface Science VI.

[18] Decher G., Hong J., Schmitt J. (1992). Buildup of ultrathin multilayer films by a self-assembly process: III. Consecutively alternating adsorption of anionic and cationic polyelectrolytes on charged surfaces. Thin Solid Films.

[19] Lvov Y., Decher G., Moehwald H. (1993). Assembly, structural characterization, and thermal behavior of Layer-by-Layer deposited ultrathin films of poly(vinyl sulfate) and poly(allylamine). Langmuir.

[20] Iler R. (1966). Multilayers of colloidal particles. J. Colloid Interface Sci..

[21] Zhao S., Caruso F., Dähne L., Decher G., De Geest B.G., Fan J., Feliu N., Gogotsi Y., Hammond P.T., Hersam M.C. (2019). The Future of Layer-by-Layer Assembly: A Tribute to ACS Nano Associate Editor Helmuth Möhwald. ACS Nano.

[22] Nunes B.N., Paula L.F., Costa Í.A., Machado A.E.H., Paterno L.G., Patrocinio A.O.T. (2017). Layer-by-layer assembled photocatalysts for environmental remediation and solar energy conversion. J. Photochem. Photobiol. C Photochem. Rev..

[23] Shi Q., Qian Z., Liu D., Liu H. (2017). Surface Modification of Dental Titanium Implant by Layer-by-Layer Electrostatic Self-Assembly. Front. Physiol..

[24] Das B.P., Tsianou M. (2017). From polyelectrolyte complexes to polyelectrolyte multilayers: Electrostatic assembly, nanostructure, dynamics, and functional properties. Adv. Colloid Interface Sci..

[25] Vilela C., Figueiredo A.R.P., Silvestre A.J.D., Freire C.S.R. (2016). Multilayered materials based on biopolymers as drug delivery systems. Expert Opin. Drug Deliv..

[26] Gomes A.P., Mano J.F., Queiroz J.A., Gouveia I.C. (2015). Layer-by-Layer Assembly for Biofunctionalization of Cellulosic Fibers with Emergent Antimicrobial Agents. Adv. Polym. Sci..

[27] Cuomo F., Lopez F., Ceglie A. (2014). Templated globules—Applications and perspectives. Adv. Colloid Interface Sci..

[28] Caruso F. (2001). Nanoengineering of Particle Surfaces. Adv. Mater..

[29] Hammond P.T. (2004). Form and Function in Multilayer Assembly: New Applications at the Nanoscale. Adv. Mater..

[30] Lutkenhaus J.L., Hammond P.T. (2007). Electrochemically enabled polyelectrolyte multilayer devices: From fuel cells to sensors. Soft Matter.

[31] Zhang X., Chen H., Zhang H. (2007). Layer-by-layer assembly: From conventional to unconventional methods. Chem. Commun..

[32] Schlenoff J.B. (2009). Retrospective on the Future of Polyelectrolyte Multilayers†. Langmuir.

[33] Guzmán E., Mateos-Maroto A., Ruano M., Ortega F., Rubio R.G. (2017). Layer-by-Layer polyelectrolyte assemblies for encapsulation and release of active compounds. Adv. Colloid Interface Sci..

[34] Holder K.M., Smith R.J., Grunlan J.C. (2017). A review of flame retardant nanocoatings prepared using Layer-by-Layer assembly of polyelectrolytes. J. Mater. Sci..

[35] Joly S., Kane R., Radzilowski L., Wang T., Wu A., Cohen R.E., Thomas E.L., Rubner M.F. (2000). Multilayer Nanoreactors for Metallic and Semiconducting Particles. Langmuir.

[36] Lee S.W., Yabuuchi N., Gallant B.M., Chen S., Kim B.-S., Hammond P.T., Shao-Horn Y. (2010). High-power lithium batteries from functionalized carbon-nanotube electrodes. Nat. Nanotechnol..

[37] Artyukhin A.B., Bakajin O., Stroeve P., Noy A. (2004). Layer-by-Layer Electrostatic Self-Assembly of Polyelectrolyte Nanoshells on Individual Carbon Nanotube Templates. Langmuir.

[38] Tang Z., Zhang Z., Wang Y., Glotzer S.C., Kotov N.A. (2006). Self-Assembly of CdTe Nanocrystals into Free-Floating Sheets. Science.

[39] Qi B., Tong X., Zhao Y. (2006). Layer-by-Layer Assembly of Two Different Polymer Micelles with Polycation and Polyanion Coronas. Macromolecules.

[40] Kim B.-S., Park S.W., Hammond P.T. (2008). Hydrogen-Bonding Layer-by-Layer-Assembled Biodegradable Polymeric Micelles as Drug Delivery Vehicles from Surfaces. ACS Nano.

[41] Cho J., Hong J., Char K., Caruso F. (2006). Nanoporous Block Copolymer Micelle/Micelle Multilayer Films with Dual Optical Properties. J. Am. Chem. Soc..

[42] Richert L., LaValle P., Payan E., Shu X.Z., Prestwich G.D., Stoltz J.-F., Schaaf P., Voegel J.-C., Picart C. (2003). Layer by Layer Buildup of Polysaccharide Films: Physical Chemistry and Cellular Adhesion Aspects. Langmuir.

[43] Chluba J., Voegel J.-C., Decher G., Erbacher P., Schaaf P., Ogier J. (2001). Peptide Hormone Covalently Bound to Polyelectrolytes and Embedded into Multilayer Architectures Conserving Full Biological Activity. Biomacromolecules.

[44] Cavalieri F., Postma A., Lee L., Caruso F. (2008). Assembly and Functionalization of DNA−Polymer Microcapsules. ACS Nano.

[45] Nam K.T., Kim D.-W., Yoo P.J., Chiang C.-Y., Meethong N., Hammond P.T., Chiang Y.-M., Belcher A.M. (2006). Virus-Enabled Synthesis and Assembly of Nanowires for Lithium Ion Battery Electrodes. Science.

[46] Hong J., Han J.Y., Yoon H., Joo P., Lee T., Seo E., Char K., Kim B.-S. (2011). Carbon-based Layer-by-Layer nanostructures: From films to hollow capsules. Nanoscale.

[47] Nakashima T., Zhu J., Qin M., Ho S., Kotov N.A. (2010). Polyelectrolyte and carbon nanotube multilayers made from ionic liquid solutions. Nanoscale.

[48] Rusling J.F., Hvastkovs E.G., Hull D.O., Schenkman J.B. (2007). Biochemical applications of ultrathin films of enzymes, polyions and DNA. Chem. Commun..

[49] Szczepanowicz K., Hoel H.J., Szyk-Warszynska L., Bielańska E., Bouzga A.M., Gaudernack G., Simon C., Warszynski P. (2010). Formation of Biocompatible Nanocapsules with Emulsion Core and Pegylated Shell by Polyelectrolyte Multilayer Adsorption. Langmuir.

[50] Sukhorukov G., Möhwald H., Decher G., Lvov Y. (1996). Assembly of polyelectrolyte multilayer films by consecutively alternating adsorption of polynucleotides and polycations. Thin Solid Films.

[51] Sukhorukov G.B., Donath E., Lichtenfeld H., Knippel E., Knippel M., Budde A., Möhwald H. (1998). Layer-by-layer self assembly of polyelectrolytes on colloidal particles. Colloids Surf. A Physicochem. Eng. Asp..

[52] Sukhorukov G.B., Donath E., Davis S., Lichtenfeld H., Caruso F., Popov V.I., Möhwald H. (1998). Stepwise Polyelectrolyte As-sembly on Particle Surfaces: A Novel Approach to Colloid Design. Polym. Adv. Technol..

[53] Cuomo F., Lopez F., Miguel M.G., Lindman B. (2010). Vesicle-Templated Layer-by-Layer Assembly for the Production of Nanocapsules. Langmuir.

[54] Cuomo F., Lopez F., Ceglie A., Maiuro L., Miguel M.G., Lindman B. (2012). pH-responsive liposome-templated polyelectrolyte nanocapsules. Soft Matter.

[55] Pinheiro A.C., Bourbon A.I., Cerqueira M.A., Maricato É., Nunes C., Coimbra M.A., Vicente A.A. (2015). Chitosan/fucoidan multilayer nanocapsules as a vehicle for controlled release of bioactive compounds. Carbohydr. Polym..

[56] Wang X., Zhao J. (2013). Encapsulation of the Herbicide Picloram by Using Polyelectrolyte Biopolymers as Layer-by-Layer Materials. J. Agric. Food Chem..

[57] Aguirre G., Ramos J., Heuts J.P.A., Forcada J. (2014). Biocompatible and thermo-responsive nanocapsule synthesis through vesicle templating. Polym. Chem..

[58] Peyratout C.S., Dähne L. (2004). Tailor-Made Polyelectrolyte Microcapsules: From Multilayers to Smart Containers. Angew. Chem. Int. Ed..

[59] Haidar Z.S., Hamdy R.C., Tabrizian M. (2008). Protein release kinetics for core–shell hybrid nanoparticles based on the Layer-by-Layer assembly of alginate and chitosan on liposomes. Biomaterials.

[60] Jo D.-G., Park J.H. (2012). Polysaccharide-Based Nanoparticles: A Versatile Platform for Drug Delivery and Biomedical Imaging. Curr. Med. Chem..

[61] Feng D., Shi J., Wang X., Zhang L., Cao S. (2013). Hollow hybrid hydroxyapatite microparticles with sustained and pH-responsive drug delivery properties. RSC Adv..

[62] Cuomo F., Lopez F., Piludu M., Miguel M.G., Lindman B., Ceglie A. (2015). Release of small hydrophilic molecules from polyelectrolyte capsules: Effect of the wall thickness. J. Colloid Interface Sci..

[63] Wågberg L., Erlandsson J. (2020). The Use of Layer-by-Layer Self-Assembly and Nanocellulose to Prepare Advanced Functional Materials. Adv. Mater..

[64] Shibraen M.H.M.A., Wang C., Yagoub H., Yuan Q., Yang S., Xu J. (2014). Interfacial complexation behavior of anionic and cationic cellulose derivatives. RSC Adv..

[65] Feitosa E., Adati R.D., Alves F.R. (2015). Thermal and phase behavior of didodecyldimethylammonium bromide aqueous dispersions. Colloids Surf. A Physicochem. Eng. Asp..

[66] Svitova T.F., Smirnova Y.P., Pisarev S.A., Berezina N.A. (1995). Self-assembly in double-tailed surfactants in dilute aqueous solutions. Colloids Surf. A Physicochem. Eng. Asp..

